# F-Box Protein FBXW17-Mediated Proteasomal Degradation of Protein Methyltransferase PRMT6 Exaggerates CSE-Induced Lung Epithelial Inflammation and Apoptosis

**DOI:** 10.3389/fcell.2021.599020

**Published:** 2021-04-20

**Authors:** Tiao Li, Xue He, Lijuan Luo, Huihui Zeng, Siying Ren, Yan Chen

**Affiliations:** ^1^Department of Respiratory Medicine, The Second Xiangya Hospital of Central South University, Changsha, China; ^2^Research Unit of Respiratory Disease, Central South University, Changsha, China; ^3^Diagnosis and Treatment Center of Respiratory Disease, Central South University, Changsha, China

**Keywords:** apoptosis, inflammation, cigarette smoke extract, protein arginine methyltransferase 6 (PRMT6), FBXW17, chronic obstructive pulmonary disease

## Abstract

Chronic obstructive pulmonary disease (COPD) is a chronic debilitating lung disease, characterized by progressive airway inflammation and lung structural cell death. Cigarette smoke is considered the most common risk factor of COPD pathogenesis. Understanding the molecular mechanisms of persistent inflammation and epithelial apoptosis induced by cigarette smoke would be extremely beneficial for improving the treatment and prevention of COPD. A histone methyl modifier, protein arginine N-methyltransferase 6 (PRMT6), is reported to alleviate cigarette smoke extract (CSE)-induced emphysema through inhibiting inflammation and cell apoptosis. However, few studies have focused on the modulation of PRMT6 in regulating inflammation and cell apoptosis. In this study, we showed that protein expression of PRMT6 was aberrantly decreased in the lung tissue of COPD patients and CSE-treated epithelial cells. FBXW17, a member of the Skp1-Cullin-F-box (SCF) family of E3 ubiquitin ligases, selectively bound to PRMT6 in nuclei to modulate its elimination in the proteasome system. Proteasome inhibitor or silencing of FBXW17 abrogated CSE-induced PRMT6 protein degradation. Furthermore, negative alteration of FBXW17/PRMT6 signaling lessened the proapoptotic and proinflammatory effects of CSE in lung epithelial cells. Our study, therefore, provides a potential therapeutic target against the airway inflammation and cell death in CS-induced COPD.

## Introduction

Chronic obstructive pulmonary disease (COPD) is a chronic debilitating and progressive lung disease, leading to more than 3 million deaths worldwide each year, which is characterized by persistent inflammation in the lung parenchyma and small airways (Lozano et al., 2012; Vogelmeier et al., 2017). Smoking is the prominent risk for the developmental progress of COPD (Shapiro, 2001; La Rocca et al., 2007; Laniado-Laborin, 2009; Angelis et al., 2014; Vogelmeier et al., 2017). Long-term exposure to cigarette smoke induces severe damage in the lung epithelial cells and contributes to their death, and thus, the induction of immune response, and subsequent destruction of lung parenchyma (Yang et al., 2006; Comer et al., 2013; Angelis et al., 2014; Kim et al., 2016). Nevertheless, progressive and persistent injury of the lungs causes irreversible airflow limitation and severely impacts a patient’s life (Vogelmeier et al., 2017). Although symptomatic treatment can attenuate symptoms and slow down the progression of disease, currently, COPD is still not curable (Lozano et al., 2012). Discovering associated signaling or key factors in immune response and cell death might provide a useful strategy for lessening the severity of pulmonary inflammation and aberrant apoptosis. Epigenetics is emerging to play an essential role in the modulation of cell fate decision and inflammatory responses, as well as in the pathogenesis of COPD (Bayarsaihan, 2011; Rivera and Ross, 2013; Wahlin et al., 2013; Hagood, 2014). Targeting epigenetic modifiers might be a potential choice for COPD therapies (Comer et al., 2015; Wu et al., 2018).

Protein arginine N-methyltransferase 6 (PRMT6) is a type I histone methyl-modified enzyme, displaying a unique substrate specificity to catalyze the asymmetric dimethylation of histone H3 arginine 2 (H3R2me2a), as well as binding to the H3 tail to prevent methylation of H3K4 (Guccione et al., 2007; Hyllus et al., 2007). PRMT6 resides predominantly in the nucleus and is associated with gene transcription suppression (Frankel et al., 2002). Despite its epigenetic function, PRMT6 also plays an essential role in the methylation of non-histone proteins and is involved in a variety of life processes, including cell senescence (Phalke et al., 2012; Stein et al., 2012), cell cycle arrest (Kleinschmidt et al., 2012; Wang et al., 2012), cell apoptosis (Kang et al., 2015; Luo et al., 2015), and immune responses (Zhang et al., 2019). Evidence shows that PRMT6 controls inflammatory gene expression via regulating transcription factors involved in inflammation and cell death signaling, including nuclear factor kappa B (NF-κB) and G-protein pathway suppressor 2 (GPS2) (Di Lorenzo et al., 2014; Zhang et al., 2019). Ablation of PRMT6 negatively regulates p53 expression and thus induces cell senescence (Neault et al., 2012; Phalke et al., 2012). Our previous work proved that PRMT6 overexpression in the respiratory system alleviated the emphysema change in a cigarette smoke extract intraperitoneal-established COPD mouse model (He et al., 2017). Collectively, these studies implicate the important role of PRMT6 in the regulation of inflammation and cell fate decision, but its modulating mechanisms in CS-induced airway epithelial cell remain unclear.

The ubiquitin–proteasome system (UPS) is a major machinery for the degradation of intracellular proteins, terminating the function of targeted proteins to regulate a multitude of cellular processes (Hershko and Ciechanover, 1986; Rock et al., 1994; Lecker et al., 2006; Schwartz and Ciechanover, 2009; Stintzing and Lenz, 2014). The enzymatic chains, including E1-activating enzyme, E2-conjugating enzyme, and E3 ligase, participate in connecting ubiquitin chains to target protein substrates (Hershko and Ciechanover, 1986; Schwartz and Ciechanover, 2009; Shen et al., 2013). The >1,000 identified E3 enzymes are the largest family, ensuring the specificity of substrate for ubiquitylation and destined degradation (Meyer-Schwesinger, 2019). Among the E3 enzymes, the SCF ligase complex is the largest and the most well-characterized subfamily (Nandi et al., 2006; Meyer-Schwesinger, 2019), while in this complex, the F-box protein (FBP) is the pivotal element for specific binding to substrate (Cardozo and Pagano, 2004). In humans, more than 60 F-box proteins have been described, which can be classified into three subunits: F-box and WD-40 domain proteins (FBXWs), F-box and leucine-rich repeat proteins (FBXLs), and F-box-only proteins (FBXOs) (Cardozo and Pagano, 2004; Skaar et al., 2013). Several FBPs, such as β-Trcp, FBXW7, FBXW15, FBXO17, and FBXL19, are characterized as participating in cellular activities (Zhao et al., 2012; Zou et al., 2013; Suber et al., 2017; Ci et al., 2018; Yang et al., 2018). However, the function of multiple FBPs still awaits further discovery. FBXW17, an ortholog of FBXW12 in mouse genome, is a 466-amino-acid protein containing an F-box motif for the recognition of substrate and the recruitment of ubiquitylation (Jin et al., 2004). As an SCF protein member, FBXW17 has a similar sequence to other FBPs, which is recently reported to ubiquitinate FBXL19 for its degradation (Dong et al., 2020). However, its function and other molecular targets remain to be determined further depending on its role in SCF subunits.

Here, we found that FBXW17 targeted PRMT6, selectively mediating the proteasomal degradation of PRMT6 to exaggerate cigarette smoke extract (CSE)-induced pulmonary inflammation and apoptosis. Our results might provide insights into developing new approaches to limit the severity of inflammation and apoptosis through regulating the proteasome machinery to dispose of indispensable epigenetic enzymes linked to CSE-induced lung injury.

## Materials and Methods

### Patients and Samples

The Research Medical Ethics Committee of the Second Xiangya Hospital of Central South University (Changsha, China) granted approval for this study. Fresh tissue samples from patients with COPD were collected from the Department of Thoracic Surgery, Second Xiangya Hospital of Central South University. Tissue samples from the lungs of patients who received thoracic surgery at the Second Xiangya Hospital were used for Western blotting.

### Cigarette Smoke Extract Preparation

Cigarette smoke extract was prepared with slight modifications, as previously described (Kang et al., 2015). Briefly, one cigarette (Furong Brand, filtered cigarettes; 12 mg tar; 1.1 mg nicotine; 14 mg carbon monoxide per cigarette) was used for bubbling through 10 ml of serum-free medium until the cigarette smoke largely disappeared in the syringe. Then, the CSE solution was filtered through an aseptic 0.22-μm filter. The filtered CSE was considered as 100% and serially diluted with culture medium for subsequent study. The CSE solution was prepared freshly for all experiments.

### Cell Culture and Reagents

Human bronchial epithelial cells (BEAS-2B) and murine lung epithelial cells (MLE-12) were, respectively, maintained with Dulbecco’s modified Eagle’s medium (DMEM)-F12 medium complemented by 10% fetal bovine serum (FBS) in a 37°C incubator with a supplement of 5% CO_2_. The cells at the fourth or fifth passage and grown to 80% confluence were used. PRMT6 (catalog no. 15395-1-AP), tumor necrosis factor (TNF)-α (catalog no. 60291-1-AP), interleukin (IL)-1β (catalog no. 16806-1-AP), and FLAG (catalog no. 66008-1-Ig) antibodies were purchased from Proteintech Group, Inc. (Chicago, IL, United States). H3R2me2a (catalog no. NB21-1002) was purchased from Novus Biologicals (Littleton, CO, United States). H3K4me3 (catalog no. 9727S), BCL2-associated X (Bax) (catalog no. 14796S), horseradish peroxidase (HRP)-conjugated anti-mouse immunoglobulin G (IgG) and HRP-conjugated anti-rabbit IgG antibodies were purchased from Cell Signaling Technology (Beverly, MA, United States). Antibodies against cyclooxygenase (COX)-2 (catalog no. ab15191) were purchased from Abcam (Cambridge, MA, United States). Immobilized protein A/G beads (catalog no. 45350) were purchased from Thermo Fisher Scientific (Waltham, MA, United States). Control IgG (sc2025) came from Santa Cruz Biotechnology (Dallas, TX, United States). Cycloheximide (catalog no. 239764) and MG-132 (catalog no. #474790) were purchased from Millipore Sigma (Burlington, MA, United States). Leupeptin (catalog no. ALX-260-009-M025) was purchased from Enzo Life Sciences (Farmingdale, NY, United States).

### Plasmid and shRNA Transfection

The plasmid vector containing V5, green fluorescence protein (GFP), or FLAG tag was constructed by and purchased from Cyagen (Shanghai, China) and Genchem (Shanghai, China). Plasmids were transfected into MLE12 cells using a Nucleofection^TM^ II system (Amaxa Biosystems, Gaithersburg, MD, United States) as previously described (Zou et al., 2013). Briefly, 1 × 10^6^ MLE12 cells were homogeneously suspended in 100 μl of electrotransfection buffer [1 × phosphate-buffered saline (PBS) with 20 mM HEPES], and 1–3 μg of plasmids was added into each cuvette. Electroporation was executed in the preset program of T-013. After electroporation, 1 ml hydrocortisone, insulin, transferrin, estradiol, and selenium (HITES) medium was immediately added to each cuvette. Transfected cells were cultured in six-well plate for 48 h and were then used for further assay. Scramble shRNA and small hairpin RNAs (shRNAs) against FBXW17 were purchased from GeneChem (Shanghai, China). shRNAs were transfected into cells by Lipofectamine 2000 reagents according to the manufacturer’s instructions. After 72 h of incubation, transfected cells were used for further CSE treatment and analysis.

### Western Blotting

Cells were washed with cold PBS and lysed with radioimmunoprecipitation assay (RIPA) buffer on ice for 30 min. After spinning down the cell-buffer mix at 14,000 g for 10 min at 4°C, an equal amount of cell lysates (20 μg) in the supernatants was subjected to sodium dodecyl sulfate–polyacrylamide gel electrophoresis (SDS-PAGE) and then transferred to a nitrocellulose (NC) membrane. Subsequently, 5% skimmed non-fat milk was used for NC membrane blocking for 1 h at room temperature and then incubated with primary antibody (PRMT6 1:1,000, H3R2me2a 1:1,000, H3K4me3 1:1,000, Bcl-2 1:1,000, Bax 1:1,000, TNF-α 1:1,000, IL-1β 1:1,000, COX-2 1:1,000, β-actin 1:5,000) at 4°C, overnight. After washing with 0.5% Tween 20 in Tris-buffered saline (TBS-T) three times, NC membranes were further incubated with HRP-conjugated secondary antibody (1:5,000–10,000) for about 1 h. Proteins were visualized using ECL reagents (Millipore, United States) and detected with an auto-radiogram system (Bio-Rad, United States).

### *In vitro* Binding Assay

MLE12 cells were lysed with an IP lysis buffer (catalog no. 87787, Thermo Fisher, United States) on ice for 30 min. Then, 1 mg of cell lysates from each group was subjected to immunoprecipitation by incubating with FLAG or PRMT6 primary antibodies overnight at 4°C, followed by incubation with 40 μl of protein A/G-agarose for 2 h at room temperature. The immunoprecipitants were then washed three times with 1% Triton X-100 in ice-cold PBS. Ladder buffer was added to resuspend beads and boiled at 95°C for 5 min. Western blotting with an enhanced ECL system was applied for detection.

### Immunofluorescence Staining

MLE12 cells (2 × 10^5^) were plated on 35 mm glass-bottom culture dishes at 70% confluence. Then, 4% paraformaldehyde was used to fix cells for a period of 20 min. Cells were then permeabilized with 0.1% Triton X-100 for 2 min. After being incubated with a 1:500 dilution of antibodies to GFP or FLAG tag, cells were immunoblotted with a 1:200 dilution of fluorescence-conjugated secondary goat anti-mouse antibody. The nucleus was stained with 4’6-diamidino-2-phenylindole (DAPI). Immunofluorescent imaging of the cell was performed on a Nikon confocal microscope.

### Quantitative Real-Time PCR

Total RNAs from cells were extracted using TRIzol reagent (catalog no. 15596026, Invitrogen, United States) and reverse transcribed into complementary DNA (cDNA) by using an RNA-to-cDNA kit (catalog no. 4387406, Applied Biosystems, United States) according to the manufacturer’s instructions. After reverse transcription, the messenger RNA (mRNA) expressions were checked by using a SYBRGREEN PCR mix (Invitrogen, United States), under the Bio-Rad CFX96 Real-Time PCR system, and β-actin was used for normalization. Primer sequences for quantitative real-time PCR are listed below: FBXW17 forward primer, 5′- ACGCCACCAGTTGAAGATGCT-3′; reverse primer, 5′-ACAGAAACCACTTCATCGGCTCT-3′; PRMT6 forward primer, 5′- CCCCGATTAGCGACCAGA-3′; reverse primer, 5′-TCTCCATGCAGCTCATATCCAC-3′; actin forward primer, 5′- CATCCTGCGTCTGGACCTGG-3′; reverse primer, 5′-TAATGTCACGCACGATTTCC-3′.

### Flow Cytometry for Apoptosis Detection

The apoptotic cells under FBXW17 overexpression and FBXW17 knockdown after CSE treatment were assessed by flow cytometry with annexin V-APC conjugate fluorescein (annexin V-APC) and propidium iodide (PI) staining. According to manufacturer’s instruction (catalog no. 640932, BioLegend, CA, United States), the cells were trypsinized and washed twice with cold BioLegend’s Cell Staining Buffer (catalog no. 420201). Then, the cells were spin down and resuspended in provided annexin V binding buffer at a concentration of 2 × 10^6^ cells/ml. After that, 100 μl of cell suspension was transferred into a 5-ml test tube, followed by adding 5 μl of annexin V-APC and 10 μl of PI solution. The cells were gently mixed and incubate in the dark for 15 min at room temperature. Finally, 400 μl of annexin V binding buffer was added to each tube. The apoptosis was analyzed by flow cytometry with Beckman CytoFLEX (Beckman Coulter, United States). The annexin V-positive, PI-negative (the lower right quadrant of the dot plot) and annexin V-positive, PI-positive (the upper right quadrant of the dot plot) cells were considered as early and late apoptotic cells, respectively. The sum of both early and late apoptotic cells percentage was assumed as apoptosis.

### Statistical Analysis

All quantified data are presented as mean ± SD. One-way analysis of variance (ANOVA) and an unpaired Student’s *t* test were used for statistical analysis. A value of *p* < 0.05 was considered indicative of statistically significant difference. All the statistical analysis was carried out with GraphPad Prism 5 software.

## Results

### CSE Diminishes PRMT6 Protein Expression

**FIGURE 1:**
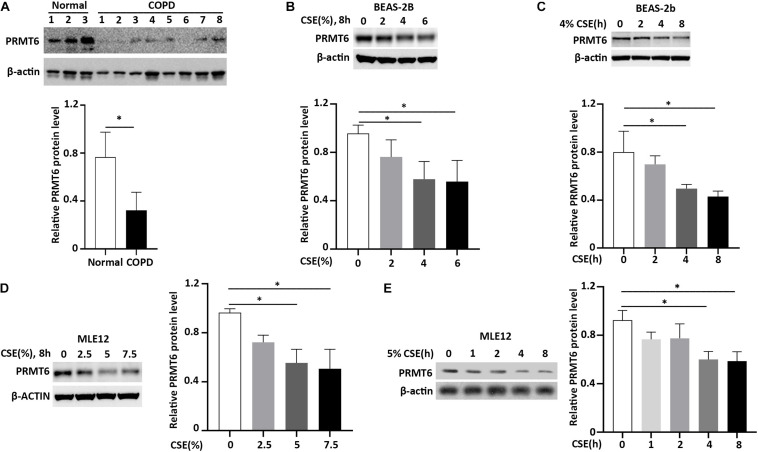
Cigarette smoke extract (CSE) diminishes protein arginine N-methyltransferase 6 (PRMT6) protein expression. **(A)** Lung tissue of chronic obstructive pulmonary disease (COPD) patients and healthy controls were lysed for PRMT6 and β-actin immunoblotting. The densitometry results of PRMT6 protein expression are plotted in the right-hand panel. **(B)** CSE (2, 4, and 6%) was applied to treat BEAS-2B cells for 8 h. Cell lysate were collected and applied to immunoblotting. The plotted densitometric results were presented in the lower panels. **(C)** BEAS-2B cells were stimulated with 4% CSE for 2, 4, and 8 h. Immunoblotting was performed to examine PRMT6 protein expression. Lower panels showed the densitometric results of the blots. **(D)** Murine lung epithelial cells (MLEs) were treated with 2.5, 5, and 7.5% CSE. **(E)** CSE (5%) was applied to MLE12 cells for a range of time points. Cell lysate was analyzed with PRMT6 or β-actin immunoblotting. The relative expression of PRMT6 protein is plotted in the right-hand panel. Data represent *n* = 3 separate experiments. The graph shows mean ± SD and “^∗^” denotes *p* < 0.05. The black line and “^∗^” indicated the differences between groups.

Accumulated data show that cigarette smoke induces lung epithelial cell dysfunction and death, following impairment to the integrity of the airway barrier contributing to the pathogenesis of COPD (Heijink et al., 2012; Amatngalim et al., 2016). PRMT6 was reported to be a negative regulator of cell apoptosis and inflammatory response (Kang et al., 2015; He et al., 2017, 2020; Zhang et al., 2019). We first evaluated the protein level of PRMT6 in the lung tissue of COPD patients with Western blotting. We observed that the protein level of PRMT6 is aberrantly decreased throughout the lung tissue of COPD patients (Figure 1A). To further verify whether cigarette smoke decreases the PRMT6 level *in vitro*, we primarily conducted CSE treatment in the human bronchial epithelial BEAS-2b cells. Results from immunoblotting analysis showed that CSE decreased PRMT6 proteins levels in both a concentration- and time-dependent manner, with 4% of CSE significantly reducing the protein levels of PRMT6 over a period of 8 h (Figures 1B,C). To confirm this observation, the mouse lung epithelial MLE12 cells were applied to CSE stimulation. In MLE12 cells, CSE with a concentration of 5% markedly reduced the PRMT6 protein level at 8 h (Figures 1D,E). These data indicated that cigarette smoke diminished PRMT6 protein in a time-dependent manner, both *in vivo* and *in vitro*.

### PRMT6 Is Degraded via Proteasome System

**FIGURE 2:**
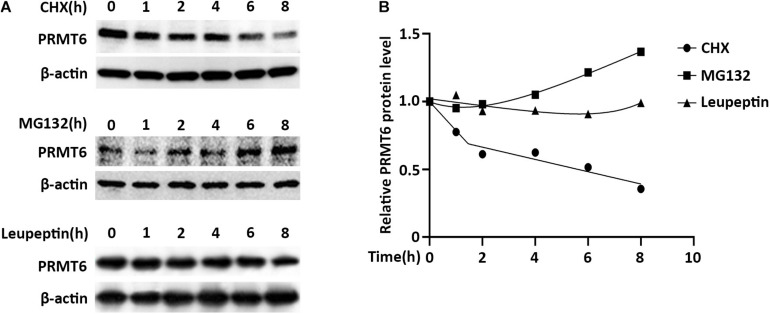
Protein arginine N-methyltransferase 6 (PRMT6) is degraded by proteasome system. **(A)** MLE12 cells were, respectively, treated with 100 μg/ml cycloheximide (CHX), 20 μM MG132, or 100 μM leupeptin at time points of 1, 2, 4, 6, and 8 h as indicated. Cell lysate was subjected to PRMT6 and β-actin immunoblotting analysis. **(B)** The densitometric changes of PRMT6 protein expression were plotted. Results are shown with a representative of *n* = 3 experiments.

Proteins destined to be turned over were generally disposed of by proteasome or lysosome machinery (Stintzing and Lenz, 2014). To investigate whether PRMT6 is unstable and supposed to be degraded, we first assessed the protein stability of PRMT6. The lung epithelial MLE cells were treated with the protein-biosynthesis inhibitor cycloheximide (CHX) (20 μg/ml), and PRMT6 protein levels were then analyzed by immunoblotting. Results showed that CHX diminished endogenous PRMT6 mass in a time-dependent manner, and PRMT6 is a liable protein with a predicted half-life (t_1/2_) of ∼5 h. To further identify whether the proteasomal or lysosomal system is involved in the degradation of PRMT6 protein, we treated MLE12 cells with proteasomal inhibitor MG-132 (20 μM) or lysosomal inhibitor leupeptin (100 μM). Endogenous PRMT6 accumulated under MG-132 exposure, while it was hardly changed under leupeptin treatment (Figures 2A,B). These results supported the belief that PRMT6 is an unstable protein degraded via proteasome machinery but not a lysosomal system.

### FBXW17 Targets the Proteasomal Degradation of PRMT6

**FIGURE 3:**
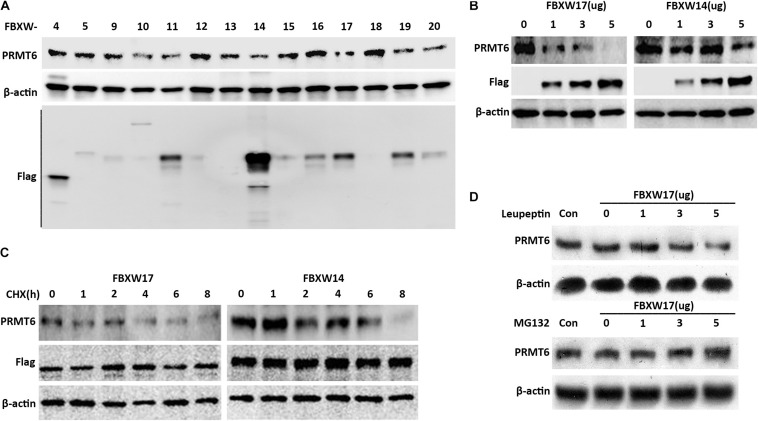
FBXW17 targets the proteasomal degradation of protein arginine N-methyltransferase 6 (PRMT6). **(A)** Each *pcDNA3.1-Flag/FBXW* plasmid as indicated were separately delivered into MLE12 cells using an electroporation program. After 48 h of transfection, the cell samples were collected and lysed; the lysates were then analyzed using PRMT6, FLAG, and β-actin immunoblotting. **(B)** Different masses of *pcDNA3.1-Flag/FBXW14* or *FBXW17* plasmids were electroporated into MLE12 cells. Cell lysates were collected and subjected to PRMT6, Flag, and β-actin immunoblotting. **(C)** Three micrograms of FBXW14- or FBXW17-overexpressed plasmids were electroporated delivered into MLE12 cells. After 48 h of transfection, the cells were treated with 100 μg/ml cycloheximide (CHX) for five time points as indicated. The immunoblotting of PRMT6, FLAG, and β-actin was tested. **(D)** The MLE12 cells were electroporated transfected with 0, 1, 3, and 5 μg of FBXW17 plasmids for 48 h. Before collection, cells were, respectively, treated with 20 μM of MG132 or 100 μM of leupeptin. Cells were harvested and lysed. Lysate was analyzed by conducting immunoblotting with the indicated antibodies. Data represent *n* = 3 separate experiments.

The important components of the SCF ubiquitin-ligase F-box proteins were used as mediators of binding to substrates for further ubiquitin–proteasomal proteolysis (Kipreos and Pagano, 2000). In order to clarify which F-box protein determined the PRMT6 degradation, several F-box-overexpressed plasmids were constructed to check the PRMT6 level. We found that FBXW17 overexpressed in MLE12 cells decreased the PRMT6 protein content (Figure 3A). To detect whether FBXW17 specifically targeted PRMT6 degradation, we randomly analyzed the effect of FBXW14-FLAG plasmid on PRMT6 degradation in MLE12 cells. Results showed that FBXW17 decreased the PRMT6 protein level in a dose-dependent manner but not FBXW14 (Figure 3B). Moreover, under the treatment of 20 μg/ml CHX, the PRMT6 protein level reduced faster in FBXW17-overexpressed cells than FBXW14-overexpressed cells (Figure 3C). To investigate whether FBXW17-mediated PRMT6 degradation is in a proteasome pathway, we overexpressed FBXW17-FLAG in wild-type MLE12 cells under MG132 or leupeptin treatment. FBXW17 decreased the PRMT6 protein level in MG132 treatment but not leupeptin (Figure 3D), indicating that FBXW17 specifically targeted PRMT6 degradation via a proteasomal pathway.

### FBXW17 Interacting With PRMT6 Colocalize in the Nucleus

**FIGURE 4:**
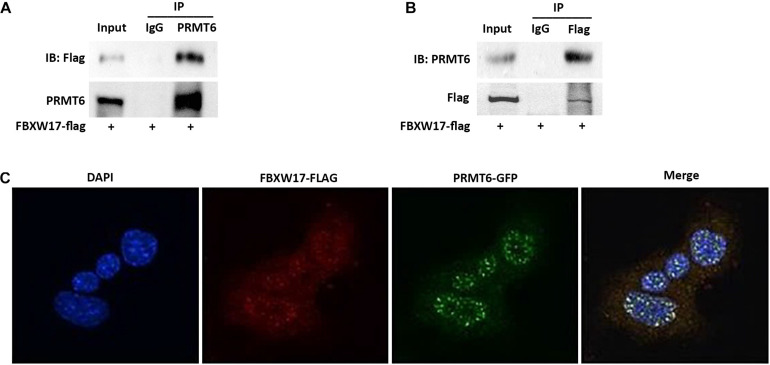
FBXW17 interacting with protein arginine N-methyltransferase 6 (PRMT6) colocalize in the nucleus. **(A)** WT FBXW17-FLAG plasmids were delivered into MLE12 cells for 48 h. After transfection, cell lysate was immunoprecipitated with PRMT6 antibody. Then, the immunoprecipitants were analyzed with FLAG and PRMT6 immunoblotting. **(B)** One microgram of cell lysate was subjected to FLAG immunoprecipitation. Immunoblotting analyses used PRMT6 antibody. **(C)** FBXW17-FLAG plasmid (red) and PRMT6-GFP plasmid (green) were cotransfected into MLE12 cells. 4′,6-Diamidino-2-phenylindole (DAPI) was used to dye the nucleus. Cells were subjected to fluorescence microscopy with different wavelengths of light. Images were merged under a confocal microscope. Results are shown with a representative of *n* = 3 experiments.

In general, targeted proteins interact with F-box proteins and are then subjected to subsequent ubiquitin–proteasomal degradation (Skaar et al., 2013). To explore whether FBXW17 directly binds with PRMT6 and mediates PRMT6 degradation, we overexpressed FLAG-tagged FBXW17 plasmids in MLE12 cells and immunoprecipitated ectopic-expressed FBXW17 protein using FLAG antibody in the presence of MG132. Further analysis of the immunoprecipitated protein mix by PRMT6 immunoblotting demonstrated that PRMT6 was associated with FBXW17 (Figure 4A). In turn, we immunoprecipitated endogenous PRMT6 by using PRMT6 antibody under MG132 stimulation and analyzed the immunoprecipitants by immunoblotting with FLAG antibody, which demonstrated that FBXW17 was also associated with PRMT6 (Figure 4B). To further elucidate the direct binding of FBXW17 and PRMT6, immunofluorescence was conducted by transfection of FBXW17-FITC (red) and PRMT6-GFP (green) plasmids. PRMT6 is considered to be a nuclear protein (Frankel et al., 2002). Our data also showed that overexpressed mouse PRMT6 with a green fluorescent protein (GFP) tag localized mainly in the nucleus. The merged figure indicated that FBXW17 and PRMT6 coexist mainly in the nucleus (Figure 4C). Overall, these results suggested that FBXW17 was associated with PRMT6 in the nucleus, indicating that FBXW17-mediated PRMT6 protein reduction might happen in the nucleus.

### FBXW17 Promotes CSE-Induced PRMT6 Degradation

**FIGURE 5:**
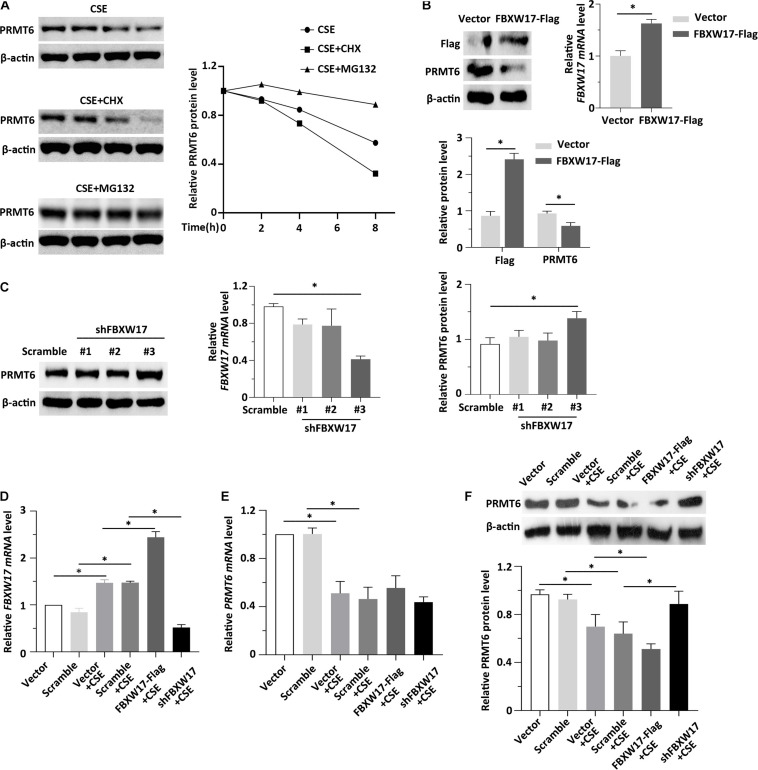
FBXW17 promotes cigarette smoke extract (CSE)-induced protein arginine N-methyltransferase 6 (PRMT6) degradation. **(A)** The MLE12 cells were stimulated with 5% of CSE and cycloheximide (CHX) or MG132. Cell lysates were subjected to immunoblotting for PRMT6 and β-actin separately. The densitometry results of PRMT6 protein expression are plotted in the right-hand panel. **(B)** MLE12 cells were transfected with vector or FBXW17 overexpression plasmids via electroporation for 48 h. The messenger RNA (mRNA) of FBXW17 was detected using quantitative real-time PCR (qRT-PCR). The data was plotted in the right panel. The relative protein expression of FLAG and PRMT6 were assayed with Western blotting. Densitometry of FLAG and PRMT6 was presented in the lower panel. **(C)** Scramble small hairpin RNA (shRNA) and three kinds of FBXW17 shRNA were delivered into MLE12 cells by using Lipofectamine 2000 reagent for 72 h. FBXW17 *mRNA* and PRMT6 protein were determined. The plotted FBXW17 *mRNA* and PRMT6 protein expression were presented in the middle panel and the right panel. **(D–F)** Vector, FBXW17-FLAG plasmid, scramble shRNA, and FBXW17 shRNA were separately transfected into MLE12 cells. Before the cells were collected, the transfected cells were stimulated with 5% CSE for 6 h. Total RNA was collected by Trizol reagents. The *mRNA* levels of **(D)** FBXW17 and **(E)** PRMT6 were determined with qRT-PCR. **(F)** Cell lysate was collected and conducted with PRMT6 or β-actin immunoblotting. The densitometric data of PRMT6 protein expression was plotted in the lower panel. Data represent *n* = 3 separate experiments. The graph shows mean ± SD, and “^∗^” denotes *p* < 0.05. The differences between each group were indicated by black line and “^∗^.”

Previous data showed that CSE decreased both the protein and mRNA level of PRMT6 (Kang et al., 2015; He et al., 2017). The levels of proteins within cells are determined not only by rates of synthesis but also by rates of degradation. However, whether CSE-mediated PRMT6 protein reduction can be blocked by proteasome inhibitor is not clear, so here, we stimulated the MLE12 cells with CSE and CHX or MG132. Notably, protein synthesis inhibitor CHX obviously promotes CSE-induced PRMT6 protein reduction, while blockade of the proteasome machinery with the proteasome inhibitor MG132 prevented PRMT6 proteins from CSE-induced degradation (Figure 5A). The above data suggested that CSE promotes PRMT6 reduction both in mRNA downregulation and protein degradation. FBXW17 mediated PRMT6 proteasomal degradation (Figures 3, 4). To determine whether FBXW17 contributed to CSE-mediated reduction in PRMT6 protein, we modified FBXW17 with overexpressed plasmids and shRNA in MLE12 cell and treated cells with CSE. RT-qPCR results showed that FBXW17 was overexpressed by FBXW17-FLAG plasmids and was successfully knocked down by shRNA (Figures 5B,C). PRM6T protein decreased under FBXW17 overexpression (Figure 5B), while it increased after FBXW17 silence (Figure 5C). Furthermore, the mRNA level of FBXW17 was upregulated by CSE (Figure 5D). Meanwhile, CSE decreased PRMT6 mRNA (Figure 5E). However, modifying FBXW17 had no influence on PRMT6 mRNA level under CSE treatment. Interestingly, silencing of FBXW17 significantly inhibited the reduction in PRMT6 protein induced by CSE; thus, overexpression of FBXW17 further promoted the protein reduction of PRMT6 in CSE-treated cells (Figure 5F). Hence, our results indicated that FBXW17 acted as an activator in CSE-induced PRMT6 protein degradation in the lung epithelial cell line.

### FBXW17/PRMT6 Signaling Involves in CSE-Induced Lung Epithelial Inflammation and Apoptosis

**FIGURE 6:**
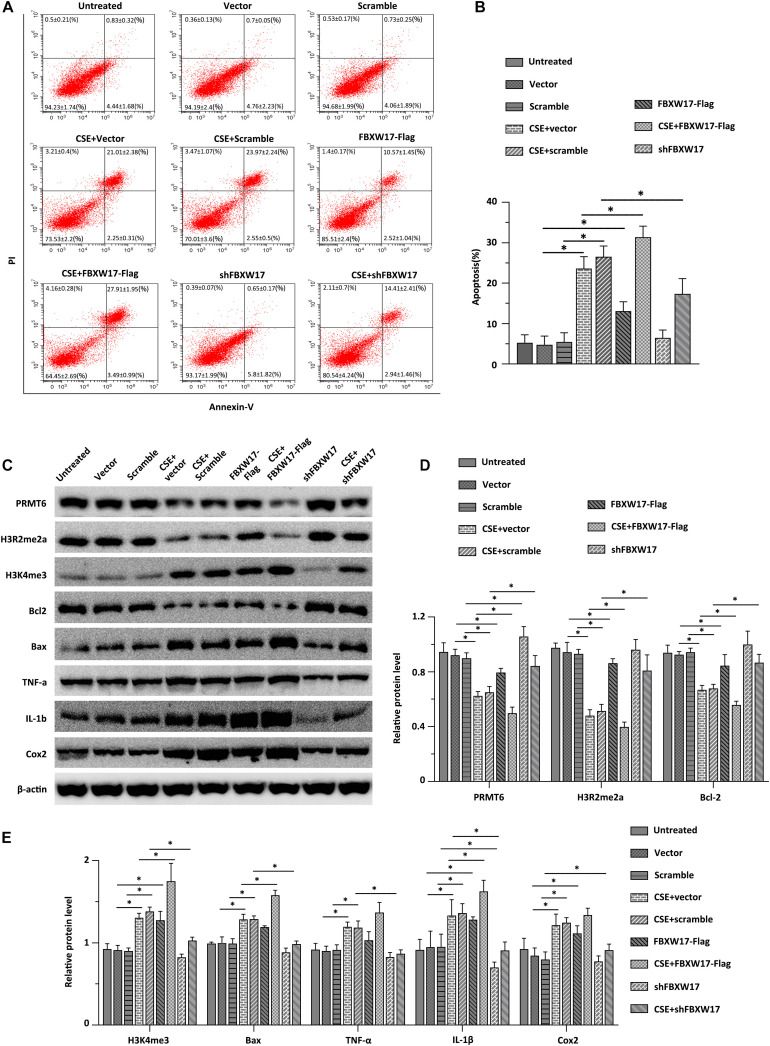
FBXW17/protein arginine N-methyltransferase 6 (PRMT6) signaling involves in cigarette smoke extract (CSE)-induced lung epithelial inflammation and apoptosis. **(A)** Vector, FBXW17-FLAG plasmids, scramble small hairpin RNA (shRNA), and FBXW17 shRNA were transfected into MLE12 cells separately. Annexin V and PI kits were used to detect the rate of apoptotic cells. Before detection, cells were treated with CSE for 6 h. The “percentage of cells ± SD” values were shown in all the quadrants of flow cytometry. **(B)** The plotted data of apoptosis in each group are presented. **(C)** MLE12 cells were transfected with either overexpressed or knockdown plasmids of FBXW17. Before collection, the cells were treated with 5% CSE for 6 h. Cell lysate was conducted with immunoblotting with indicated antibodies. **(D)** The plotted PRMT6, H3R2me2a, and Bcl-2 protein expression were shown. **(E)** The relative protein expression of H3K4me3, Bax, TNF-α, IL-1β, and COX-2 were presented. Data were represented by *n* = 3 separate experiments. The graph shows mean ± SD. “^∗^” denotes *p* < 0.05 between indicated groups.

Cigarette smoke induced aberrant activation of proteasome (Kim et al., 2011). F-Box proteins were suggested to be enrolled in cigarette smoke-stimulated protein degradation, indicating the potential roles of F-box protein in COPD development (Mallampalli et al., 2020). Combined with the above data, we hypothesized that CSE-induced lung inflammation and apoptosis may be regulated by FBXW17/PRMT6 signaling. We first tested whether overexpression of FBXW17 affected the apoptosis of lung epithelial cells. In line with our above observations of FBXW17 increasing in CSE-treated cells (Figure 5D), ectopic expression of FBXW17 increased the apoptosis rate of MLE12 cells (Figure 6A, the middle panel). Given the detrimental effect on cell apoptosis, we further evaluated the effect of FBXW17 silencing on CSE-induced epithelial cell apoptosis. As expected, FBXW17 knockdown partly alleviated CSE-induced lung epithelial apoptosis (Figure 6A, the lower panel). PRMT6 is suggested to target H3 histone arginine (R2) and cause asymmetric dimethylation of R2 (H3R2me2a), thereby inhibiting the occurrence of gene transcription (Hyllus et al., 2007). As previous studies showed, PRMT6 inhibits inflammation and apoptosis in endothelial cells with decreased methylation of the histone H3R2 site (Kang et al., 2015). Our data also proved that CSE reduced the PRMT6 level and H3R2me2a signal, as well as elevating proapoptotic protein Bax and inflammatory factors in lung epithelial cells (Figures 6C–E). In addition, the CSE-regulated PRMT6 level and its downstream signals were modified by FBXW17. Overexpression of FBXW17 enhanced the reduction in PRMT6 and the H3R2me2a signal in CSE treatment, accompanied by increased Bax and COX-2 expression (Figure 6E). On the other hand, suppression of FBXW17 enhanced the inhibitory effects of PRMT6 and H3R2me2 on inflammation and apoptosis in lung epithelial cells induced by CSE (Figure 6D). Collectively, these data indicated that FBXW17/PRMT6 signaling plays an important role in regulating CSE-induced lung epithelial inflammation and apoptosis.

## Discussion

In this study, we identified that (i) PRMT6 protein decreased in COPD; (ii) cigarette smoke extract decreased the unstable protein PRMT6 in epithelial cells; (iii) FBXW17 interacted with PRMT6 and specifically mediated the proteasomal degradation of PRMT6 protein in the nucleus; (iv) inhibition of the proteasome pathway and knockdown of FBXW17 partly blocked CSE-induced PRMT6 protein reduction; and (v) FBXW17/PRMT6 signaling was involved in CSE-induced lung epithelial inflammation and apoptosis. Noxious particles, including cigarette-smoke-induced airway inflammation, epithelial cell apoptosis, and oxidative stress, are considered to be the initial steps in COPD development (Tzortzaki and Siafakas, 2009). In addition, long-term exposure to cigarette smoke induces remodeling and narrowing of small airways and further causes the consequent destruction of the lung parenchyma as indicated by losing the alveolar attachments as a result of emphysema, and finally progression to airflow limitation in the lung, which is the main pathological progression of COPD (Shapiro and Ingenito, 2005). Epigenetic modifications and related enzymes are believed to be involved in COPD pathogenesis. The most confirmed epigenetic enzyme in COPD is histone deacetylase 2 (HDAC2), and its reduction is considered to be linked to airway inflammation amplification and glucocorticoid resistance in COPD treatment (Barnes, 2009; Lai et al., 2018). Cigarette smoke dysregulates epigenetic enzymes modulates inflammatory responses, thus participating in the progression of chronic respiratory disease (Wu et al., 2018; Zong et al., 2019). Our previous work has proved that cigarette smoke reduces the histone arginine methyltransferase PRMT6 protein level in vascular endothelial cells (Kang et al., 2015). Moreover, ectopic PRMT6 expression in the lung alleviated emphysema morphology change in a CSE-established mouse model (He et al., 2017, 2020). In this study, we add another paradigm of the fact that PRMT6 decreased in COPD and CSE-stimulated lung epithelial cells, indicating its potential role in CSE-mediated inflammation and cell apoptosis in epithelial cellular models.

Aberrant expression of PRMT6 widely exists in cancers like lung (Avasarala et al., 2020), liver (Chan et al., 2018), gastric (Okuno et al., 2019), and prostate (Vieira et al., 2014) cancer and viral infections (Zhang et al., 2019). However, the modulation of PRMT6 protein has not been elucidated. Several histone modification enzymes are naturally subject to degradation, but whether it occurs through ubiquitin–proteasomal machinery, specifically mediated by F-box components, is more limited (Zou and Mallampalli, 2014). For the first time, we reported that PRMT6 is a short-life protein and is degraded in a proteasome pathway specifically mediated by FBXW17 F-box protein, instead of lysosome. Coimmunoprecipitation of PRMT6 and FBXW17 showed that ectopically expressed F-box subunit FBXW17 coexisted with PRMT6 and resulted in PRMT6 degradation. Frankel et al. (2002) showed that PRMT6 resides predominantly in the nucleus. Immunofluorescent staining of PRMT6 in our study also showed that it mainly existed in the nucleus and followed the ectopically expressed of FBXW17. Moreover, CSE resulting in PRMT6 protein reduction was partly rescued by proteasome inhibitor of MG132 or FBXW17 knockdown. FBXW17-induced PRMT6 turnover also caused a shift of histone arginine methylation, thus influencing the inflammatory and apoptotic gene transcription. Although others have shown that FBXW17 is restricted to mouse genome, our data raised a new paradigm for the activation of the FBXW17 gene specifically within lung epithelia by CSE to control histone modification and related enzymes.

F-Box proteins contribute to the pathway of ubiquitin-mediated protein disposal by specifically providing substrate to the SCF superfamily of E3 ligases (Cardozo and Pagano, 2004; Meyer-Schwesinger, 2019). SCF complex-mediated protein degradation has been proven to participate in numerous cellular activities, such as gene expression regulation and signaling transduction (Skowyra et al., 1997, 1999). Accumulated data have shown that F-box proteins play important roles in the modulation of inflammation or cell apoptosis. Zhao et al. (2012) reported that F-box protein FBXL19 mediates the ubiquitin–proteasomal degradation of the indispensable receptor linked to interleukin 33 (IL-33) to limit sepsis-induced pulmonary inflammation. A recent research showed that FBXW17 ubiquitinates FBXL19 for its degradation. Silence of FBXW17 attenuated lysophosphatidic acid (LPA)-induced epithelial cell migration (Dong et al., 2020), which suggested a potential role of FBXW17 in cell function regulation. Small molecules inhibiting FBXO3 reduce cytokine release and lessen associated inflammatory diseases via destabilizing tumor necrosis factor receptor-associated factor (TRAF) protein (Chen et al., 2013; Mallampalli et al., 2013). In lung epithelia, FBXO17 targets glycogen synthase kinase-3β (GSK3β) for its polyubiquitination and degradation, thus limiting LPS-induced inflammation (Suber et al., 2017). In addition, FBPs govern cell apoptosis via targeting BCL-associated proteins for degradation (Inuzuka et al., 2011; Duan et al., 2012). Here, we showed that FBXW17 overexpression exaggerated CSE-induced epithelial cell apoptosis and inflammation through repressing the antiapoptotic BCL-2 protein while promoting the proapoptotic protein Bax and inflammatory genes like IL-1β and COX-2. Moreover, silencing of FBXW17 ameliorated smoke-induced apoptosis and inflammation of epithelia. Our study highlighted the importance of manipulating the proteolytic processing of a methyltransferase by F-box protein for proteasomal degradation, which was suggested to profoundly attenuate CSE-mediated lung epithelial inflammation and cell apoptosis. Our research also added a paradigm of FBXW17 function in cell fate decision.

In summary, these findings identify that inhibiting FBXW17/PRMT6 signaling is an important way to attenuate cigarette-smoke-induced inflammation and apoptosis of epithelial cell. This study also elucidates a novel molecular mechanism of PRMT6 regulation and reveals a crosstalk between epigenetic modification and proteasome system regulation.

## Data Availability Statement

The raw data supporting the conclusions of this article will be made available by the authors, without undue reservation.

## Ethics Statement

The studies involving human participants were reviewed and approved by the Research Ethics Committee of the Second Xiangya Hospital of Central South University. The patients/participants provided their written informed consent to participate in this study.

## Author Contributions

YC was responsible for the study concept and design. TL, XH, and LL performed the experiments. TL, HZ, and SR contributed to analysis and interpretation of the data and responsible for critically revising the manuscript. All authors involved in editing the manuscript and approved the final version for submission.

## Conflict of Interest

The authors declare that the research was conducted in the absence of any commercial or financial relationships that could be construed as a potential conflict of interest.
